# Fumigant Toxicity of Oriental Sweetgum (*Liquidambar orientalis*) and Valerian (*Valeriana wallichii*) Essential Oils and Their Components, Including Their Acetylcholinesterase Inhibitory Activity, against Japanese Termites (*Reticulitermes speratus*)

**DOI:** 10.3390/molecules190812547

**Published:** 2014-08-19

**Authors:** Il-Kwon Park

**Affiliations:** Department of Forest Science, Research Institute of Agriculture and Life Science, College of Agriculture and Life Sciences, Seoul National University, Seoul 151-921, Korea; E-Mail: parkik1@snu.ac.kr; Tel.: +82-2-880-4751; Fax: +82-2-880-3560

**Keywords:** fumigant toxicity, plant essential oils, oriental sweetgum, valerian, Japanese termites, acetylcholinesterase inhibitiory activity

## Abstract

This study investigated the fumigant toxicity of oriental sweetgum (*Liquidambar orientalis*) and valerian (*Valeriana wallichii*) essential oils and their components against the Japanese termite (*Reticulitermes speratus*). The fumigant toxicity of oriental sweetgum and valerian oil differed significantly according to exposure time. Oriental sweetgum showed toxicity at short exposure times (2 days), and the toxicity of valerian oil was high 7 days after treatment. The main constituents of oriental sweetgum and valerian oils were tested individually for their fumigant toxicity against Japanese termites. Among the test compounds, benzyl alcohol, acetophenone, 1-phenyl-1-ethanol, hydrocinnamyl alcohol, *trans*-cinnamyl aldehyde, *trans*-cinnamyl alcohol, *cis*-asarone, styrene, and *cis*-ocimene showed toxicity against Japanese termites 7 days after treatment. Hydrocinnamyl alcohol and *trans*-cinnamyl alcohol were found to be the major contributors to the fumigant antitermitic toxicity of oriental sweetgum oil. The acetylcholinesterase (AChE) inhibition activity of two oils and their constituents was tested to determine their mode of action. Only *cis*-ocimene showed strong AChE inhibition activity with an IC_50_ value of 0.131 mg/mL. Further studies are warranted to determine the potential of these essential oils and their constituents as fumigants for termite control.

## 1. Introduction

Termites cause more than $3 billion in damage to wooden structures annually throughout the U.S. [1]. Because termite damage is hard to detect, wooden structures are already seriously damaged from the inside when surface changes emerge. Among the 2800 described species of termites, about 185 are considered economically important pests [2].

The Japanese termite (*Reticulitermes speratus* Kolbe) is the one termite species described in Korea, which recently has caused serious damage to wooden structures such as temples and palaces [3,4,5,6]. The main control methods for the Japanese termite in Korea are application of synthetic pesticides or wood preservatives, which are effective, but cause side effects such as environmental pollution, human health problems, toxicity to natural enemies, and development of resistance. To reduce the side effects of synthetic pesticides, we urgently need to develop alternative methods for termite management. Plant extracts or essential oils could be good candidates to replace synthetic pesticides and wood preservatives. Many plant extracts or essential oils have insecticidal or repellent effects against termites, as Verma *et al.* have summarized [2]. Plant essential oils often show fumigant toxicity or repellent effects against termites. If proper formulations were available, they could be used as fumigants for quarantine or applied on the wood surface to repel termites. Furthermore, plant essential oils evaporate easily, therefore, residues are not a problem [7,8,9].

In this study, we investigated the fumigant toxicity of essential oils from oriental sweetgum (*Liquidambar orientalis*) and valerian (*Valeriana wallichii*). We also determined the fumigant toxicity of essential oil components and their acetylcholinesterase (AChE) inhibitory activity to determine if their mode of action could be attributed the inhibition of this enzyme.

## 2. Results and Discussion

### 2.1. Chemical Components of Plant Essential Oils

The chemical analysis of oriental sweetgum and valerian essential oils was well described in our previous study [10], and is summarized in Table 1. The main components of oriental sweetgum oil were *trans*-cinnamyl alcohol (45.07%) followed by hydrocinnamyl alcohol (41.13%), β-caryophyllene (3.6%), styrene (1.56%), benzyl alcohol (1.22%), and α-pinene (1.02%). The composition ratios of other compounds were all less than 1%. The most abundant compound of valerian oil was *cis*-asarone (88.82%), and the composition ratios of *trans*-asarone, linalool, and *cis*-ocimene were 3.41%, 0.13% and 0.12%, respectively.

**Table 1:** Chemical composition ^a^ of oriental sweetgum and valerian essential oils.

No.	Compound	Standard Compound Purity, Company ^b^	Retention Indices		Composition Ratio (%, w/w)	
			DB-1MS	DB-FFAP	Oriental Sweetgum	Valerian
1	Styrene	95%, T	875	1257	1.56	-
2	α-Pinene	95%, T	929	1014	1.02	-
3	Benzaldehyde	99%, A	929	1526	0.47	-
4	β-Pinene	94%, T	968	1100	0.15	-
5	Benzyl alcohol	99%, W	1007	1880	1.22	-
6	*cis*-Ocimene	90%, S ^c^	1027	Nd^c^	-	0.12
7	Acetophenone	98%, T	1033	1659	0.19	-
8	1-Phenyl-1-ethanol	99%, S ^d^	1034	1815	0.17	-
9	Linalool	98%,W	1088	1536	-	0.13
10	Hydrocinnamyl alcohol	98%, S ^d^	1205	2049	41.13	-
11	*trans*-Cinnamyl aldehyde	99%, A	1230	2049	0.24	-
12	*trans*-Cinnamyl alcohol	98%, A	1279	2273	45.07	-
13	β-Caryophyllene	90%, T	1415	1587	3.60	-
14	*cis*-Asarone	70%, A	1586	2312	-	88.82
15	*trans*-Asarone	98%, A	1639	2413	-	3.41
	Sum	-	-	-	94.82	92.48

^a^ Chemical analysis of oriental sweetgum and valerian oils has been reported in our previous study [10]. ^b^ A (Aldrich), S ^c^ (SAFC), S ^d^ (Synthesized in our laboratory), T (TCI), and W (Wako). ^c^ Not detected.

### 2.2. Antitermitic Activity of Plant Essential Oils and Individual Compounds

The antitermite activity of plant essential oils and their components against Japanese termites is shown in Table 2 and Table 3. Oriental sweetgum essential oil showed 100% fumigant toxicity against Japanese termites 2 and 7 days after treatment at 1.2 mg/L air. Valerian oil exhibited moderate or weak fumigant toxicity 2 days after treatment, but showed 100% fumigant toxicity 7 days after treatment over the whole range of concentrations tested. This result indicates that exposure time is an important factor when evaluating the fumigant toxicity of plant essential oils. High vapor pressure of the fumigant is needed to kill the pests. Phytochemicals from plant essential oils have varying rates of vapor pressure [11]. Vapor pressure of *cis*-asarone is 1.3 × 10^−3^ mm Hg at 20 °C [12], and this low vapor pressure might be one reason why valerian oil and *cis*-asarone show weak fumigant toxicity at 2 days after treatment. Among the essential oil components, benzyl alcohol, acetophenone, 1-phenyl-1-ethanol, hydrocinnamyl alcohol, and *trans*-cinnamyl aldehyde exhibited strong fumigant toxicity against Japanese termites 2 days after treatment at 2.5 mg/L air. The fumigant toxicity of *trans*-cinnamyl alcohol was 100% at 10 mg/L air, but it was reduced to 36% at 5 mg/L air. The other compounds showed moderate or weak toxicity 2 days after treatment at 10 mg/L air. The fumigant toxicity of essential oil components 7 days after treatment is shown in Table 3. Benzaldehyde, benzyl alcohol, aceptophenone, 1-phenyl-1-ethanol, hydrocinnamyl alcohol, *trans*-cinnamyl aldehyde, *trans*-cinnamyl alcohol, and *cis*-asarone showed 100% toxicity against Japanese termites at 1.25 mg/L air. The fumigant toxicities of styrene and *cis*-ocimene were 94% and 80% at 5 mg/L air, but they were reduced to 4% and 14% at 2.5 mg/L air, respectively. The fumigant toxicity of *trans*-asarone was very weak 7 days after treatment at a high concentration. The fumigant toxicities of α-pinene, β-pinene, linalool and β-caryophyllene were not tested in this study, because their toxicities against Japanese termites were reported in our previous study [5,6]. Seo *et al.* [5] reported that the phenol, alcohol, and aldehyde groups were more toxic to Japanese termites than the hydrocarbon group. That was also observed in this study. The fumigant toxicities of styrene and *cis*-ocimene which belong to the hydrocarbon group, were much weaker than those of the alcohol, aldehyde, and ketone groups. Another common structural feature of the active compounds (benzaldehyde, benzyl alcohol, acetophenone, 1-phenyl-1-ethanol, hydrocinnamyl alcohol, *trans*-cinnamyl aldehyde, *trans*-cinnamyl alcohol, and *cis*-asarone) is the presence of a benzene ring. This result indicates that a benzene ring is closely related to the insecticidal activity of compounds active against Japanese termites. Lee *et al.* [13] and Choi *et al.* [14] insisted that the antifungal and nematicidal activity of primary alcohol was stronger than that of secondary and tertiary alcohols. However, such a tendency was not observed in this or our previous study [5]. A significant difference in bioactivity, such as insecticidal and nematicidal activity between *cis*-*trans* diastereomers of asarone has been well described in previous studies [10,15,16]. The insecticidal activity of *cis*-asarone was much stronger than that of *trans*-asarone against adult insect pests such as *Sitophilus oryzae*, *Callosobruchus chinensis*, and *Lasioderma serricorne* [15]. There was a significant difference in the insecticidal activity of *cis*- and *trans*-asarone against *Nilaparuvata lugens* and *Plutella xylostella* [16]. Kim *et al.* [10] reported that the nematicidal activity of *cis*-asarone was much stronger than that of *trans*-asarone. In this study, a similar result was observed, although a long exposure time was necessary for *cis*-asarone to show fumigant toxicity against Japanese termites. Previous and present studies insist that the geometrical structure of asarone is essential in insecticidal or nematicidal activity.

**Table 2:** Fumigant antitermitic activity of oriental sweetgum and valerian essential oils and their components against Japanese termites at 2 days.

Compounds	Mortality (%, Mean ± S.E., N = 5)					
	10 ^a^	5	2.5	1.25	0.62	0.31
Oriental sweetgum	100a ^b^	100a	100a	100a	10 ± 17.3cd	0b
Valerian	52 ± 22.8b	42 ± 21.6b	24 ± 11.4bc	20 ± 15.8de	14 ± 16.7cd	6 ± 8.9b
Styrene	54 ± 11.4b	48 ± 8.3b	2 ± 4.4cd	-	-	-
Benzaldehyde	100a	100a	100a	38 ± 10.95cde	16 ± 20.7cd	4 ± 5.4b
Benzyl alcohol	100a	100a	100a	62 ± 31.1abc	46 ± 18.1bc	20 ± 15.8b
*cis*-Ocimene	54 ± 15.1b	36 ± 16.7b	10 ± 12.2bcd	-	-	-
Aceptophenone	100a	100a	100a	88 ± 13.0ab	82 ± 13.0ab	14 ± 8.9b
1-Phenyl-1-ethanol	100a	100a	100a	100a	66.8 ± 8.9ab	22 ± 16.4b
Hydrocinnamyl alcohol	100a	100a	94 ± 5.4a	54 ± 16.7bcd	10 ± 7.0cd	-
*tans*-Cinnamyl aldehyde	100a	100a	100a	100a	100a	100a
*trans*-Cinnamyl alcohol	100a	36 ± 16.7b	28 ± 13.0b	22 ± 16.4cde	10 ± 12.2cd	8 ± 13.0b
*cis*-Asarone	52 ± 21.6b	24 ± 11.4bc	22 ± 16.4bcd	20 ± 15.8de	14 ± 11.4cd	6 ± 8.9b
*trans*-Asarone	2 ± 4.4c	- ^c^	-	-	-	-
Control	0c	0c	0d	0e	0d	0b
	F_13,__56_ = 69.96 *p* < 0.0001	F_12,__52_ = 74.21 *p* < 0.0001	F_12,__52_ = 168.71 *p* < 0.0001	F_10,__ 44_ = 33.49 *p* < 0.0001	F_10,__ 44_ = 33.89 *p* < 0.0001	F_9,__ 40_ = 46.25 *p* < 0.0001

^a^ mg/L air. ^b^ Means within a column followed by the same letters are not significantly different (Scheffé’s test). ^c^ Not tested.

**Table 3:** Fumigant antitermitic activity of oriental sweetgum and valerian essential oils and their components against Japanese termites at 7 days.

Compounds	Mortality (%, Mean ± S.E., N = 5)					
	10 ^a^	5	2.5	1.25	0.625	0.31
Oriental sweetgum	100a ^b^	100a	100a	100	56 ± 19.4bc	6 ± 8.9cd
Valerian	100a	100a	100a	100	100a	100a
Styrene	96 ± 8.9a	94 ± 8.9ab	4 ± 5.4bc	-	-	-
Benzaldehyde	100a	100a	100a	100	34 ± 20.7cd	6 ± 5.4cd
Benzyl alcohol	100a	100a	100a	100	86 ± 19.4ab	32 ± 8.3b
*cis*-Ocimene	80 ± 21.1a	80 ± 21.1b	14 ± 15.1b	-	-	-
Aceptophenone	100a	100a	100a	100	100a	16 ± 11.4bcd
1-Phenyl-1-ethanol	100a	100a	100a	100	98 ± 4.4a	24 ± 16.7bc
Hydrocinnamyl alcohol	100a	100a	100a	100	24 ± 18.1cd	-
*tans*-Cinnamyl aldehyde	100a	100a	100a	100	100a	100a
*trans*-Cinnamyl alcohol	100a	100a	100a	100	46 ± 5.4c	14 ± 5.4bcd
*cis*-Asarone	100a	100a	100a	100	100a	100a
*trans*-Asarone	4 ± 8.9b	- ^c^	-	-	-	-
Con	0b	0c	0c	0	0d	0d
	F_13,__56_ = 142.77 *p* < 0.0001	F_12,__52_ = 94.17 *p* < 0.0001	F_12,__52_ = 426.97 *p* < 0.0001	-	F_10,__ 44_ = 47.86 *p* < 0.0001	F_9,__ 40_ =145.91 *p* < 0.0001

^a^ mg/L air. ^b^ Means within a column followed by the same letters are not significantly different (Scheffé’s test). ^c^ Not tested.

### 2.3. Fumigant Toxicities of Artificial Blends

**Figure 1:**
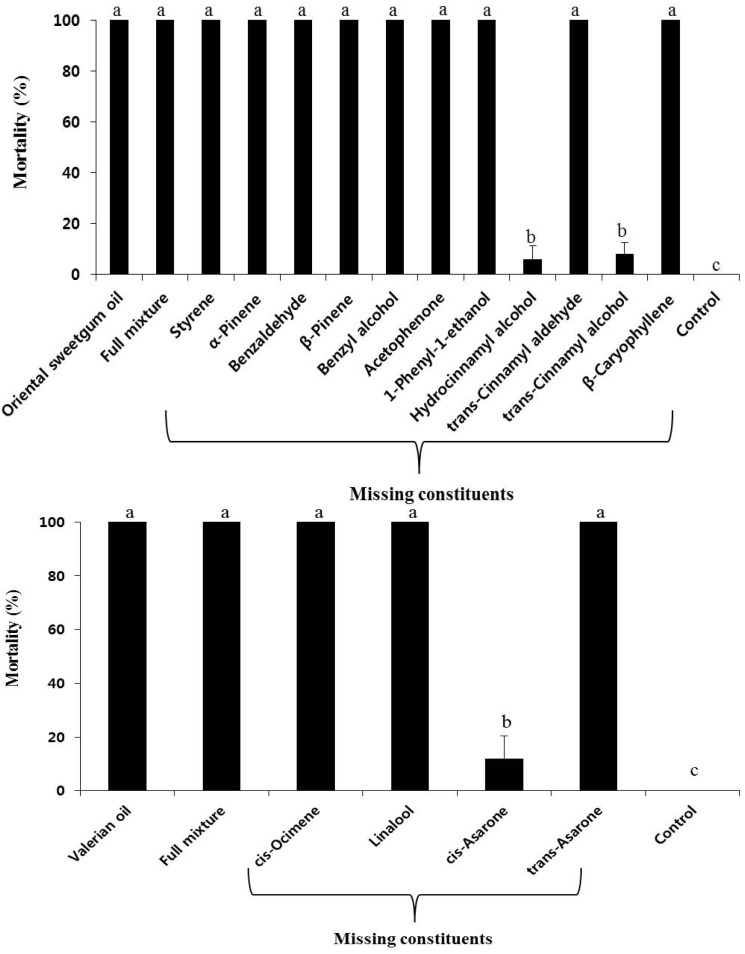
Fumigant toxicities of oriental sweetgum, valerian oil, a full mixture, and selected blends of the constituents in Japanese termite adults 7 days after treatment. The concentrations of oriental sweetgum and valerian oil were 2.5 and 10 mg/L air, respectively. The concentrations of the full mixture of oriental sweetgum and valerian oil were 2.37 and 9.24 mg/L air, respectively. The concentrations of the other blends were determined by removing each constituent equivalent to the ratio identified in oriental sweetgum oil. Mean values corresponding to each treatment with different letters are significantly different from each other (oriental sweetgum oil: F_13,__56_ = 250.66, *p* < 0.0001; valerian oil: F_6,__28_ = 1057.90, *p* < 0.0001, Scheffé’s test).

The fumigant toxicities of artificial blends of oriental sweetgum and valerian oils are shown in Figure 1. Oriental sweetgum oil and a full mixture containing 11 known constituents showed 100% fumigant toxicity against Japanese termites 7 days after treatment at 2.5 mg/L air (Figure 1). There was no significant difference in fumigant toxicity between oriental sweetgum oil and artificial mixtures containing all the constituents (Figure 1, F_15,__ 64_ = 256.41, *p* < 0.0001). A constituent elimination test of oriental sweetgum oil indicated that the exclusion of hydrocinnamyl alcohol and *trans*-cinnamyl alcohol caused a significant drop in the fumigant toxicity of the blend (F_15,__ 64_ = 256.41, *p* < 0.0001). Therefore hydrocinnamyl and *trans*-cinnamyl alcohol are major contributors to the fumigant toxicity of oriental sweetgum oil, and the two compounds act synergistically in their insecticidal activity against Japanese termites. The exclusion of other compounds showed no significant difference in fumigant toxicity against Japanese termites. In the case of valerian oil, *cis*-asaone was the major contributor to the fumigant toxicity (F_6,__28_ = 1057.90, *p* < 0.0001). Knowing the role of individual components of certain plant essential oils in insecticidal, nematicidal or antifungal activity could give us important information on the standard ratio of each component when we commercialize plant essential oils as pesticides.

### 2.4. Primary AChE Inhibition Assay and IC_50_ Estimation

**Figure 2:**
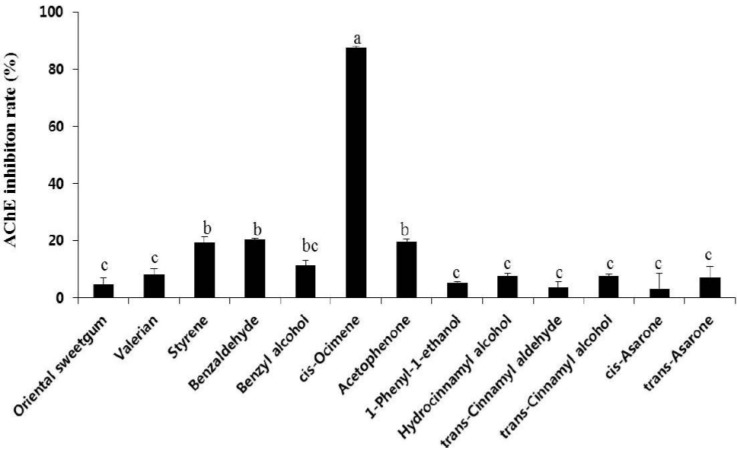
Japanese termiteacetylcholinesterase inhibition rates of oriental sweetgum, valerian oil and their constituents at 1 mg/mL. Mean values corresponding to each treatment with different letters are significantly different from one another (F_12,__26_ = 290.1, *p* < 0.0001, Scheffé’s test).

We tested the Japanese termite acetylcholinesterase (AChE) inhibition of oriental sweetgum and valerian oils and their constituents, and the primary inhibition rates of test compounds are shown in Figure 2. Among the test oils and compounds, *cis*-ocimene showed the highest inhibition rate (87.5%), followed by benzaldehyde (20.2%), acetophenone (19.5%), styrene (19.4%), and benzylalcohol (11.3%). The inhibition rates of two oils and the other constituents were less than 10%. The half minimal inhibitory concentration (IC_50_) value of *cis*-ocimene was 0.131 mg/mL (slope = 1.41 ± 0.14; 95% confidence limit = 0.105–0.160; χ^2^ = 1.72). The fumigant toxicity of *cis*-ocimene was not strong compared to the other active compounds except at a higher concentraton (10 mg/L air). This indicated that the fumigant toxicity of *cis*-ocimene might be related to the inhibition of AChE in Japanese termites. The Japanese termite adult AChE inhibition rates of α-pinene, β-pinene, linalool and β-caryophyllene were reported in our previous study [17].

The AChE inhibition activity of chemicals from plant essential oils has been well described in insects and nematodes [18,19,20,21,22]. Yeom *et al.* [18,22] reported that α-pinene, carvacrol, dihydrocarvone and isoeugenol inhibited AChE in German cockroaches. Kim *et al.* [21] tested the inhibition activity of components identified in Apiaceae essential oils against *Sitophilus oryzae* AChE. α-Pinene revealed the highest inhibition rate followed by β-pinene and limonene. Several monoterpenoids and aliphatic compounds were tested for their AChE inhibition activity against pine wood nematode [19,20]. In this study, not all compounds with strong fumigant toxicity against Japanese termites showed AChE inhibition activity. This result indicates that those chemicals have different target sites. Other than AChE, few studies have been reported on the mode of action of phytochemicals, even though various oils or their constituents cause symptoms that suggest a neurotoxic mode of action [23]. Enan insisted that insecticidal activity of eugenol was attributed to its binding to octopamine receptors [24]. Price and Berry reported that geraniol and citral showed some simililarities to octopamine [25]. Lei *et al.* asserted that nematicidal activity of thymol and carvacrol was mediated through a tyramine receptor (TyrR) [26]. However, the exact mode of action of essential oils or their constituents is still not completely understood.

## 3. Experimental Section

### 3.1. Termites

In 2012 and 2013, several pine logs were buried in the soil at Hongneung Arboretum, Seoul (37°35'N, 127°02'E), Korea, to collect termites. The termite-infested pine logs were then moved to the laboratory, moistened with distilled water, and kept in plastic cages (60 × 40 × 40 cm) at 24–26 °C and 60% relative humidity.

### 3.2. Plant Essential Oils and Chemicals

Plant essential oils were purchased from AYUS GmbH (Weinstrasse, Bühl/Baden, Germany). Information about the chemicals used in this experiment is given in Table 1. 1-Phenyl-1-ethanol and hydrocinnamyl alcohol (3-phenylpropan-1-ol) were synthesized in our laboratory as explained in Kim *et al.* [10]. 

### 3.3. Instrumental Analysis

The chemical analysis of oriental sweetgum and valerian essential oils was accomplished as described in our previous study [10]. In brief, Agilent 6890N gas chromatograph (GC) instrument (Agilent Technologies, Santa Clara, CA, USA) equipped with an FID detector was used to analyze the oriental sweetgum and valerian oils. Retention times to compare the standard compounds were obtained using DB-1MS and DB-FFAP column (column length: 30 m, internal diameter: 0.25 mm, film thickness: 0.25-μm; J&W Scientific, Folsom, CA, USA). The initial oven temperature was 40 °C for 1 min, which was then elevated to 250 °C at the rate of 6 °C/min, and kept at the final temperature for 4 min. The carrier gas was helium, and the flow rate was 1.5 mL/min. An Agilent 6890N GC coupled with a 5973N mass selective detector (MSD) was used for analysis of the two oils using a DB-5MS (30 m × 0.25 mm i.d., 0.25-μm film thickness; J&W Scientific) column. The oven temperatures were same as those used for GC analysis. The injection weight of the oils was 1 μg.

### 3.4. Antitermitic Activity

Fumigant toxicity against Japanese termites was evaluated using a glass cylinder (diameter: 5 cm, height: 10 cm) with a wire sieve installed about 5 cm above the bottom. The essential oils or their components were applied to a paper disc (8 mm, Advantec, Tokyo, Japan). The treated paper disc was laid on the bottom lid of the glass cylinder, and the lid was sealed with parafilm to prevent the essential oils or their components from leaking. We transferred ten adult worker termites to the wire sieve, which prevented the direct contact between termites and the test oils or their compounds. Filter paper fully sprayed with water was used for food. Control termites were supplied with filter paper only. The test termites were kept at 25 ± 1 °C and 60% relative humidity. Cumulative mortalities of termites were investigated 2 days and 7 days after treatment. All treatments were replicated five times.

### 3.5. Fumigant Toxicities of Artificial Blends

To determine the contribution of each component from oriental sweetgum oil to its fumigant toxicity against Japanese termites, we made a blend that mimicked the natural oil. We also prepared several blends of oil constituents, each lacking 1 constituent (Figure 1). Blends were based on the natural composition rate of oriental sweetgum oil, as analyzed by GC-FID (Table 1). In a fumigant toxicity test, the concentrations of oriental sweetgum oil and the full mixture of artificial oriental sweetgum oil were 2.5 mg/L air and 2.37 mg/L air, respectively. We determined the concentrations of other blends by eliminating each component equivalent to the ratio identified in oriental sweetgum oil. We also contrasted the toxicities of the complete and incomplete blends with that of oriental sweetgum oil.

### 3.6. Primary AChE Inhibition Assay and IC_50_ Estimation

We extracted the crude protein from 50 termites together using a glass tissue grinder (Wheaton Industries Inc., Millville, NJ, USA). First, the Japanese adult termites were dipped in 300 µL 0.1 M Tris-HCl (pH 7.8) containing 20 mM NaCl, 0.5% Triton X-100, and a protease inhibitor cocktail (Sigma-Aldrich, St. Louis, MO, USA) and ground using a glass tissue grinder in ice. The crude extract was centrifuged at 15,000× *g* for 15 min at 4 °C, and the protein (supernatant) was used for the AChE inhibition test. The concentration of crude protein was measured by Bradford protein assay and BSA was used as the standard protein. The AChE inhibition activity was measured using the modified Ellman’s method [27]. The 11 compounds tested in this study were prepared by diluting them in acetone to 100 mg/mL. We mixed 1 μL of test compounds and 79 μL crude protein (10 mg) in 96-well microplates and incubated them for 10 min at room temperature. Acetone without test compounds was used as a positive control. After pre-incubation, 10 µL of 10 mM acetylthiocholine iodide (ATCH) and 10 µL of 4 mM 5,5'-dithiobis (2-nitrobenzoic acid) (DTNB) were added to the mixture of test compounds and crude protein. The AChE was observed by estimating the initial velocity (V_o_) for 30 min at 30-second intervals at 405 nm at RT using an iMark microplate absorbance reader (Bio-Rad, Hercules, CA, USA). The AChE inhibition activity was estimated using the following equation:

Inhibition activity (%) = 100 − (V_o_ of chemical treatment/V_o_ of control treatment × 100)

*cis*-Ocimene which showed >50% AChE inhibition activity against the crude protein of termites, was chosen, and its AChE inhibition activity was measured at five concentrations (1, 0.5, 0.2, 0.1, and 0.05 mg/mL) to obtain the IC_50_ value. IC_50_ was estimated using probit analysis [28]. All AChE inhibition assays were replicated three times.

### 3.7. Statistical Analysis

Mortality was transformed to arcsine square root values for analysis of variance (ANOVA). Treatment means were compared and separated by Scheffé’s test [28].

## 4. Conclusions

Our results indicate that oriental sweetgum and valerian essential oils and their constituents could be useful for managing Japanese termites. However, further study is essential to determine toxicity to humans, develop proper formulations, and reduce costs before oriental sweetgum and valerian oils and their components can find practical use as novel fumigants. 
